# FeCo: Hysteresis, Pseudo-Critical, and Compensation Temperatures on Quasi-Spherical Nanoparticle

**DOI:** 10.3390/nano15050320

**Published:** 2025-02-20

**Authors:** Julio Cesar Madera, Elisabeth Restrepo-Parra, Nicolás De La Espriella

**Affiliations:** 1 Grupo Teseeo, Facultad de Ciencias e Ingenierías, Departamento de Ciencias Básicas, Universidad del Sinú-Elías Bechara Zainúm, Monteria 230001, Colombia; jmadera@unisinu.edu.co; 2 Facultad de Ciencias Básicas, Departamento de Física y Electrónica, Universidad de Córdoba, Monteria 230002, Colombia; 3 PCM Computational Applications, Facultad de Ciencias Básicas, Departamento de Física, Universidad Nacional de Colombia, Manizales 170001, Colombia; erestrepopa@unal.edu.co

**Keywords:** FeCo nanoparticles, hysteresis, compensation temperature, pseudo-critical temperatures

## Abstract

We investigated the hysteresis, pseudo-critical, and compensation behaviors of a quasi-spherical FeCo alloy nanoparticle (2 nm in diameter) using Monte Carlo simulations with thermal bath-type algorithms and a 3D mixed Ising model. The nanostructure was modeled in a body-centered cubic lattice (BCC) through the following configurations: spin S=3/2 for Co and Q=2 for Fe. These simulations reveal that, under the influence of crystal and magnetic fields, the nanoparticle exhibits compensation phenomena, exchange bias, and pseudo-critical temperatures. Knowledge of this type of phenomena is crucial for the design of new materials, since compensation temperatures and exchange bias improve the efficiency of advanced magnetic devices, such as sensors and magnetic memories. Meanwhile, pseudo-critical temperatures allow the creation of materials with controlled phase transitions, which is vital for developing technologies with specific magnetic and thermal properties. An increase in single-ion anisotropies within the nanosystem leads to higher pseudo-critical and compensation temperatures, as well as superparamagnetic behavior at low temperatures.

## 1. Introduction

Complex nanomagnetic structures exhibit diverse physical behaviors when subjected to the influence of internal and external interactions. These behaviors are reflected in some phenomena, such as first- and second-order transitions, compensation points, and exchange anisotropies [1,2]. In this work, a nanoparticle structured by body-centered cubic (BCC) lattices that is type B2 ferrimagnetic of FeCo intermetallic alloys [3] and encapsulated in a spherical geometry nanoconfiguration of 2 nm in diameter is considered. The problem to be solved in this research consists in the thermomagnetic characterization of this nanosystem, through an analysis of the hysteresis, pseudo-critical, and compensation behaviours that it exhibits. The system has been considered an approximate concentration of 50/50, with spin values S=±3/2,±1/2 for Co and Q=±2,±1,0 for Fe. This interesting alloy of Fe and Co can be chemically disordered from its equiatomic phase B2 in bulk into an A2-type phase for temperatures of the order of 730 °C [4]. When the alloy is ordered (phase B2), the crystal behaves as the intercalation of two simple cubic sublattices, one of Fe and the other of Co; this is unlike phase A2, where the atoms of the compound are randomly distributed [5].

The importance of this research lies in the fact that, according to the composition of the FeCo binary intermetallic alloy, the alloy has demonstrated useful properties such as a high maximum value of saturation magnetization (up to 240 emu/g in bulk), low single-ion anisotropies, high permeability along with good mechanical properties, and high temperatures of second-order transitions [5]. Among the applications to which the aforementioned properties give rise, we could highlight potential uses of FeCo alloys in biomedicine through magnetic resonance imaging [6,7], as well as for anticancer therapies [8,9]. Similarly, FeCo is used in magneto-elastic soft actuators [10,11], high-frequency power applications [12,13], electromagnetic wave absorption [14,15], and high technology applications such as magnetic recording media, and the generation of permanent magnets through exchange coupled nanocomposites with hard/soft magnetic phases [16]. Studies by Vadillo et al. reported the synthesis of FeCo nanoparticles in the BCC structure using the chemical reduction technique, highlighting its importance in the fabrication of magnetorheological fluids (RFMs) [17].

Scientific work at nanometer scales in magnetic materials has allowed the discovery of novel phenomena and physical properties not seen at larger scales, which has generated expectations for new applications in different fields of engineering and technology. The FeCo binary alloy is no stranger to this type of research in the field of nanostructures, which can be corroborated in the works of Kolhatkar et al., Dalavi et al., and Kandapallil et al., who showed saturation magnetization in the range from 100 to 220 emu/g for FeCo nanoparticles synthesized by chemical methods [18,19,20]. Likewise, Kodama et al. reported disordered cubic FeCo nanostructures at room temperature after being synthesized by polyol processes and with high-spin moments [21]. With this same process, Karipoth et al. report the physical properties of flower-like ${\mathrm{Fe}}_{50}{\mathrm{Co}}_{50}$ nanoparticles prepared by a unique polyol process based on the addition of precursors at an elevated temperature [22]. An additional interesting aspect investigated for FeCo nanoparticles is their usefulness in high-frequency electrical circuits due to their magnetization rapidly changing direction once the magnetic field is reversed, as well as the property of being magnetically soft [23]. There is also the use of FeCo magnetic nanoalloys in the heating properties for magnetic fluid hyperthermia [24]. Comparable in interest to what this research has stated is the research of Kim et al., who developed a two-step electro-less plating method to control the morphology and grain size of FeCo films to obtain excellent magnetic properties [25]; this goes alongside the works of Sánchez et al. on the structural analysis and magnetic properties of FeCo alloys obtained by mechanical alloying [26] and Saber et al. and their studies of the magnetic properties of the ternary ${\mathrm{FeCo}}_{x}{\mathrm{Ni}}_{1-x}$ alloy with mixed spins Fe (*S* = 2), Co (σ = 3/2) and Ni (*Q* = 1) using Monte Carlo simulations and the Metropolis Algorithm [2].

To solve the problem at hand, we will be employing computational simulations with Monte Carlo techniques (MC), thermal bath-type algorithms, and a mixed Ising 3D model. These theoretical models have been widely used to explain the qualitative behavior of various spin configurations, given their complementarity with experimental studies, helping to predict thermomagnetic properties and to understand the relevant underlying mechanisms [27,28,29]. The versatility of the Ising model allows for constructing the Hamiltonian, which defines the system under study by adding different interactions that enable understanding the role of each of them in the thermomagnetic behavior of the system [30]. The remainder of this paper is organized as follows. In Section 2, we formulate the model and describe briefly the Monte Carlo simulations. In Section 3, we analyze the results, and finally, in Section 4, we discuss them.

## 2. Model and Computational Method

### 2.1. Model

**Figure 1:**
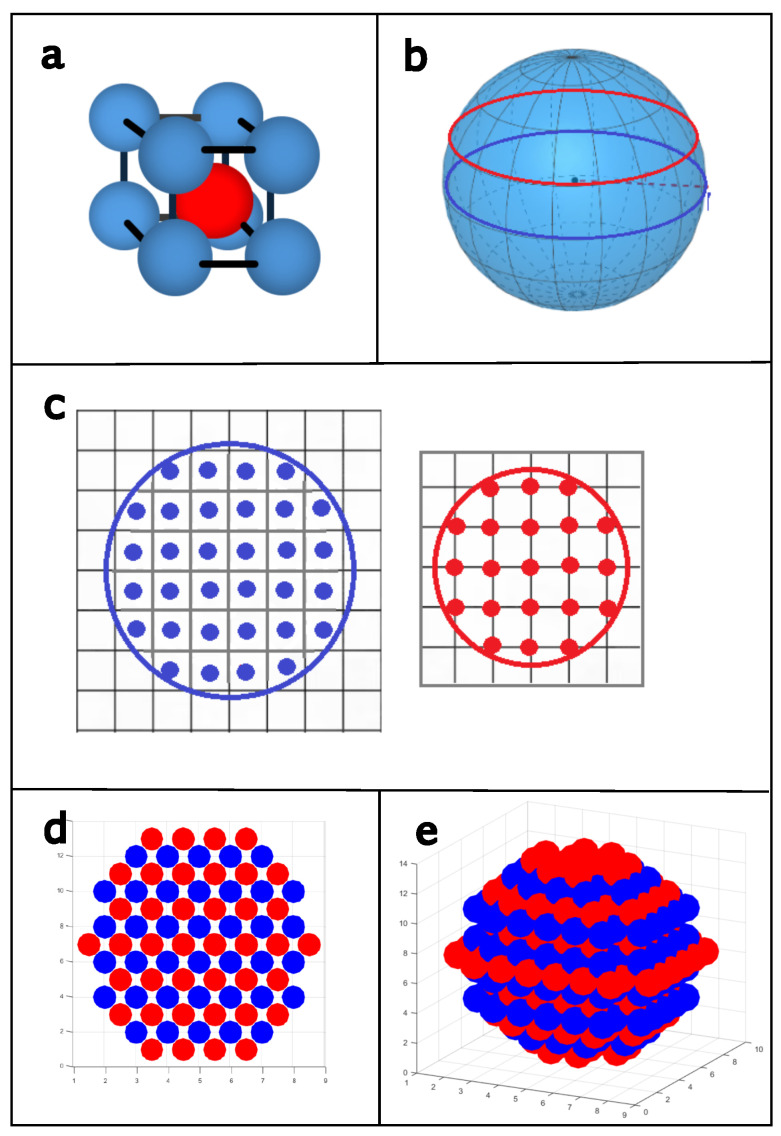
Schematic representation of FeCo quasi-spherical nanoparticle. (**a**) BCC structure of type B2; (**b**) nanoparticle geometry; (**c**) cross-sections of Fe and Co atoms; (**d**) surface view; (**e**) isometric view of quasi-spherical nanoparticle.

The system under study is a nanoparticle with a quasi-spherical geometry of 2 nm, which is constituted by body-centered cubic structures, which are of type B2 of ferrimagnetic FeCo alloys; the system has an approximate concentration of 50/50, with spin values of S=±3/2,±1/2 for Co in a sublattice *A* of size $L_{A}$ and Q=±2,±1,0 for Fe in a sublattice *B* of size $L_{B}$ (Figure 1a). The numerical modeling of the nanoparticle was performed by dividing the sphere into cross-sections, which were separated according to the lattice parameters of the BCC structure for the FeCo alloy (Figure 1b). Each slice is a circular plane with different diameters according to its location on the sphere, containing the positions of the Fe (red) and Co (blue) spins (Figure 1c). In one slice, the spins of one type of atom are located in the interstitial spaces; in the following slice, the other type is located in the vertices of the unit cell (Figure 1d). The ensemble of all planes forms the quasi-spherical nanoscaled structure (Figure 1e). Based on the above, the nanoparticle capacity was structured with 487 Ising-type atoms, 243 Co and 244 Fe.

When the alloy is ordered (phase B2), the crystal behaves as the intercalation of two simple cubic sublattices *A* and *B* of spins S=3/2 (Co) and Q=2 (Fe), respectively. For its thermomagnetic characterization, we consider antiferromagnetic exchange interactions with the next neighbors (〈nn〉) in the lattice and anisotropic couplings of the *A* and *B* sublattices, as well as interactions with an external magnetic field. Free boundary conditions on the surface atoms will be used in the simulation. The interactions of the system are defined through the following Hamiltonian:

(1) $$\begin{array}{ccc} H & = & -J_{1}\sum_{\langle nn\rangle }S_{i}^{A}Q_{j}^{B}-D_{1}\sum_{i\epsilon A}{\left(S_{i}\right)}^{2}-D_{2}\sum_{j\epsilon B}{\left(Q_{j}\right)}^{2}-h\sum_{i\epsilon A}S_{i}-h\sum_{j\epsilon B}Q_{j} \end{array}$$

In Equation (1), $J_{1}<0$ represents 〈nn〉 ferrimagnetic isotropic bilinear exchange interactions ($S_{i}^{A}\to Q_{j}^{B}$). $D_{1}$ and $D_{2}$ represent anisotropic biquadratic single-ion couplings in the *A* and *B* sublattices, and *h* is an applied external field. $D_{1}$, $D_{2}$, and *h* act on each of the ising-type atoms in the sublattices of the ferrimagnet. All the parameters are in energy units. We define dimensionless parameters reduced as follows: $D_{1}^{\prime }=D_{1}/\left|J_{1}\right|$, $D_{2}^{\prime }=D_{2}/\left|J_{1}\right|$, $h^{\prime }=h/\left|J_{1}\right|$, and $T^{\prime }=k_{B}T/\left|J_{1}\right|$. $k_{B}$ is the Boltzmann constant.

### 2.2. Monte Carlo Simulations

Ferrimagnetic nanomagnetic systems are complex spintronic structures due to the effects of size, shape, and interactions within the material. The (δ)-dimensions of the nanostructures are in the 1⩽δ⩽100 range [31], which allows them particular physical properties, unlike larger magnetic configurations; the origin of these properties is a result of changes in the electronic levels and the increase in surface atoms with respect to bulk atoms. Spintronic nanoconfigurations are catalogued as a link between bulk materials and atomic structure; thus, researching this type of material proves fascinating. All of the above characteristics make it very complex to analyze a nanoscaled material. Therefore, appropriate experimental and/or theoretical techniques must be employed. In this work, Monte Carlo simulations will be used; such methods have proven to be a useful tool for the magnetic characterization of these types of systems [32,33]. The computational technique is executed as follows: Once the initial configuration is organized, the following ones are randomly selected and changed through the Boltzmann probability. The transition probabilities are then calculated, considering that the number of possible changes depends on the type of particle located in the lattice. The data were obtained with $2\times 10^{6}$ Monte Carlo steps per site, initially discarding the first $10^{4}$ steps per spin, until equilibrium was reached. Errors were calculated by the block method, dividing the sample of size Monte Carlo into *b* blocks of size $T_{b}=L/b$, where *L* is the total sample. This implies that the errors are estimated from the block averages and not from the original measurements. Error bars are calculated by grouping all measurements in 10 blocks and taking their standard deviation [34,35].

The total ($M_{T}$) and sublattice ($M_{A},M_{B}$) magnetization and total magnetic susceptibility ($\chi_{T}$) were calculated with the following expressions:

(2) $$M_{A}=\frac{2}{L_{A}^{2}}\langle \sum_{i}S_{i}^{A}\rangle \;M_{B}=\frac{2}{L_{B}^{2}}\langle \sum_{j}Q_{j}^{B}\rangle$$

(3) $$M_{T}=\frac{1}{2}\left(M_{A}+M_{B}\right)$$

(4) $$\chi_{T}=\frac{\beta }{L^{2}}\left\{\langle M_{T}^{2}\rangle -{\langle M_{T}\rangle }^{2}\right\}\;$$

considering that 〈H〉 represents the internal energy of the system and $L=L_{A}+L_{B}$, and where $\beta =1/k_{B}T$. Due to the size of the quasi-nanosphere, the location of the transition temperature is considered a pseudo-critical temperature $\left(T_{pc}\right)$, located in a range where the magnetic susceptibility presents a maximum of smooth or “flattened” contour. To locate the compensation points experienced by the nanosystem, the intersection point of $M_{T}$ with the temperature axis was found.

## 3. Results

Thermomagnetic characterization of the nanospherical ferrimagnetic system was determined by the anisotropic effects of the crystal fields of the *A* and *B* sublattices, as well as by the influence of external magnetic fields on the magnetic moments S=±3/2,±1/2 for Co and Q=±2,±1,0 for Fe.

### 3.1. $J_{1}$–$D_{1}^{\prime }$–$D_{2}^{\prime }$ Model: Effects of $D_{1}^{\prime }$

**Figure 2:**
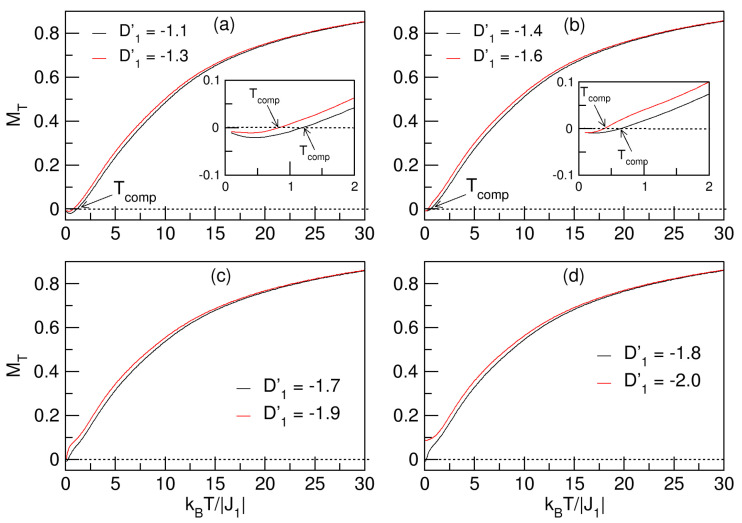
Magnetization behavior of the quasi-spherical FeCo nanosystem. The insets in (**a**,**b**) show the compensation points ($T_{comp}$) experienced by the nanoparticle in the ranges $-1.6⩽D_{1}^{\prime }⩽-1.1$ and $D_{2}^{\prime }=1$. In (**c**,**d**), it is observed that the negative increase in $D_{1}^{\prime }$ destroys the compensation phenomenon.

$D_{1}^{\prime }$ represents the magnetocrystalline anisotropy experienced by the S=3/2 spin lattice of Co and the $J_{1}$–$D_{1}^{\prime }$–$D_{2}^{\prime }$ model indicates that no magnetic field is applied to the nanoparticle ($h^{\prime }=0$). We will analyze the effects of the $D_{1}^{\prime }$ field on the $M_{T}$ magnetization and the total magnetic susceptibility $\chi_{T}$ when $J_{1}=-1$ and $D_{2}^{\prime }=1$. In Figure 2a–d, the behavior of $M_{T}$ as a function of temperature in the range $-2⩽D_{1}^{\prime }⩽-1.1$ is analyzed.

The nanosystem undergoes compensation phenomena at low temperatures when $-1.7⩽D_{1}^{\prime }$, which are denoted as $T_{comp}$, and are enlarged in the insets of Figure 2a,b. The compensation points are generated by the struggle between the anisotropic fields, because $D_{1}^{\prime }<0$ helps to keep the lattice spins with the antiferromagnetic orientation exerted by the $J_{1}$ exchange; also, $D_{2}^{\prime }=1$, being of lower intensity, exerts a slight opposition to try to direct the magnetic moments in the opposite direction. The competition between the crystal fields of the sublattices arises because $D_{1}^{\prime }<0$ implies that the spin states of the *A* sublattice (*S*-spin Co atoms) have a preference to orient themselves toward the hard-to-magnetize axis, and $D_{2}^{\prime }>0$ entails that the *B* sublattice (*Q*-spin-Fe atoms) orients itself toward the easy axis. Moreover, since the spin states Q=±2 are larger than S=±3/2, the system is compensated up to a certain value of $D_{1}^{\prime }$(−1.7,−1.1), and for values smaller than −1.7, the lattice decompensates. Next, for $k_{B}T/\left|J_{1}\right|⪆1.25$, the temperature increase destroys the compensation in the sublattices, with the $k_{B}T$ thermal energy prevailing over the effects of the crystal fields. In the $M_{T}$ curves, the temperatures of the transition of the system to the paramagnetic phase are not clearly visible due to size effects.

We consider that the effect of not observing the critical temperatures, despite the nanosphere not yet being subjected to external fields, is due to factors present in the nanoscaled systems, such as the following: **(i)** The *coordination number (z)* of the lattice that composes them. In this case, it is a BCC (z=8) with antiferromagnetic couplings 〈nn〉, implying a competition of the atoms to align themselves antiparallel in the crystal, possibly leading to the non-achievement of minimum energy configurations for all pairs of atoms at the same time—that is, a phenomenon of magnetic frustration could occur, since there can be several configurations of analogously low energy. **(ii)** The second factor has to do with *the various types of interactions* in the Hamiltonian, such as $J_{1}$ bilinear exchange and ($D_{1}^{\prime },D_{2}^{\prime }$) intrinsic anisotropic biquadratic couplings in the sublattices, with opposite orientations. **(iii)** A third aspect for the non-appearance of the critical temperature in the $M_{T}$ of the nanosphere is the existence of pseudo-critical temperatures ($T_{pc}$); these are within a range surrounding the ferri-paramagnetic phase transition with some features analogous to the critical transition, without clearly defining the transition to the paramagnetic state. In this case, we can state that the interactions considered for the quasi-spherical nanoparticle do not allow the system to be configured with a clear and specific magnetic order. **(iv)** Finally, the nanoparticle is of diameter 2 nm; it is thus small enough not to neglect anisotropic effects on the surface, which can affect the spintronic configuration of the core (innermost spins) [36].

On the other hand, it should be noted that when working with very small nanoparticles, the so-called critical size ($S_{c}$) must be taken into account, below which it can happen that the nanostructure has a single domain and can become a uniformly magnetized core, while having its net spin oriented along one of its easy magnetocrystalline directions. In this case, to demagnetize the nanoparticle, an external field must be applied that overcomes the anisotropy of the system and causes a rotation of the coupling generated by the exchange interaction. Otherwise, if we have a nanoparticle with size larger than $S_{c}$, a multidomain nanoscale particle, the energy barrier to overcome is considered relatively low and the nanosystem could be magnetized or demagnetized with an external field that moves the domain walls [37].

**Figure 3:**
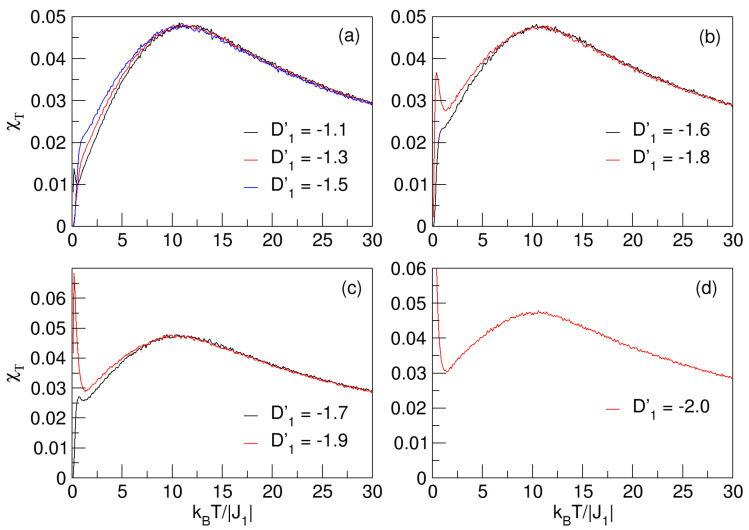
Response function of magnetic fluctuations of quasi-spherical FeCo nanoparticle $\left(\chi_{T}\right)$ varying with temperature. Curves (**a**,**b**) present flatter maxima, which increase slightly with negative decrease in $D_{1}^{\prime }$ in ranges $-1.8⩽D_{1}^{\prime }⩽-1.1$ and $D_{2}^{\prime }=1$. Curves of $\chi_{T}$ in (**c**,**d**) show same behavior in ranges $-1.7⩽D_{1}^{\prime }⩽-2$ and $D_{2}^{\prime }=1$. $\chi_{T}$ maxima represent pseudo-critical temperatures.

A way to approximately establish the location of pseudo-critical temperatures in nanostructured materials is by analyzing the magnetic fluctuations resulting from interactions in the nanosystem. In Figure 3, this analysis is performed through the total susceptibility $\chi_{T}$.

In each of the $\chi_{T}$ curves, there is notably a non-sharp maximum that is quite “flattened”, meaning the temperature for the transition to the disordered phase could not be determined accurately. In this case, critical phase transitions cannot be referred to, but pseudo-critical ones can because of their anomalous and significant effects in some physical properties, such as magnetization and its response function $\chi_{T}$, causing internal restructuring of the material in a significant way. It is important to mention that the nanosize of the quasi-sphere (2 nm), imposes certain spatial constraints, i.e., a kind of magnetic confinement, which influences the $J_{1}$, $D_{1}^{\prime }$, and $D_{2}^{\prime }$ interactions, hence the thermomagnetic characteristics of the nanoparticle. For instance, the nanoconfiguration will have more magnetic stability at higher temperatures compared to bulk materials, indicating that the system experiences less impact on its thermomagnetic fluctuations and less influence on its magnetic order. The minor effect on thermal fluctuations can cause momentary rearrangements between different magnetic states of insufficient intensities to generate a transition that is not very clear, but is still significant in the production of pseudo-critical transition temperatures. Similarly, the so-called confinement, due to size effects, can lead to a reduction in the intensity of the exchange coupling and to a more localized distribution of magnetic moments; in addition, it is able to generate an anisotropic increase in the sublattices, which could be reflected in the coercivity of the system. The $\chi_{T}$ curves in Figure 3 reflect a more gradual transition, which is not abrupt as in bulk systems, indicating that the ferri-paramagnetic transition is smoothed because of the size; further, confinement effects remove the critical energy that gives rise to a sudden transition—hence, these temperatures are considered pseudo-critical ($T_{pc}$). No less important in the study of pseudo-critical transitions is the effect of negative anisotropies on the thermomagnetic behavior of nanosubstances. Similar is the case of $D_{1}^{\prime }<0$, wherein it may happen that the magnetization is aligned on a plane that is perpendicular to a specific axis of axial direction, such that the spin orientations minimize the energy in that plane—i.e., the spin moments are aligned perpendicular to the plane where the energy is lowest, which may lead to thermal destabilization of the nanosystem, reflected, for example, in its hysteretic behavior and in the magnetic domains that may arise. In this case, the influences of $D_{1}^{\prime }<0$ would make the XY plane the difficult magnetization axis and the model could be easier magnetized through the Z axis.

### 3.2. $J_{1}$–$D_{1}^{\prime }$–$D_{2}^{\prime }$ Model: Effects of $D_{2}^{\prime }$

**Figure 4:**
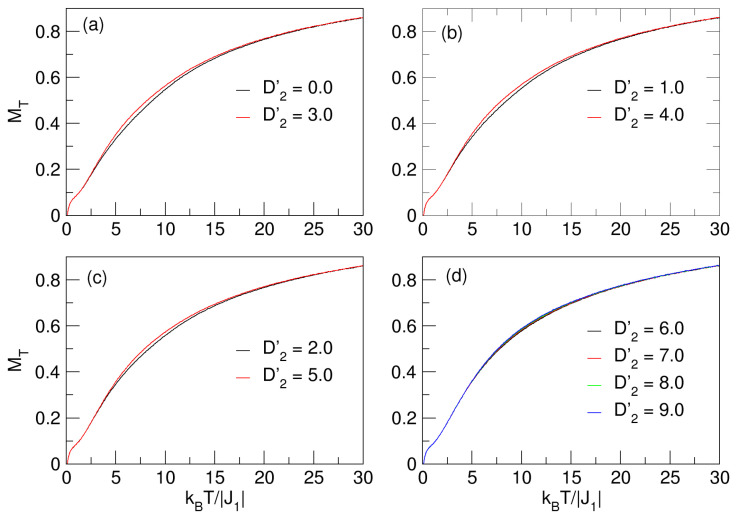
Behavior of the total magnetization of the quasi-spherical FeCo nanosystem, due to the effects of the $D_{2}^{\prime }⩾0$ and $D_{1}^{\prime }=-1.9$ fields. In the curves of (**a**–**d**), $M_{T}$ increases with temperature. No compensation points were found for this case.

Next, Figure 4 shows the behavior of the nanosphere magnetization under the effects of the crystal field $D_{2}^{\prime }⩾0$ in the interval $0⩽D_{2}^{\prime }⩽9$, for $D_{1}^{\prime }=-1.9$. We can observe that the qualitative behavior of $M_{T}$ is similar to the effects caused by $D_{1}^{\prime }<0$ on the system, both in magnitude and in the shape of the curves, as evidenced in Figure 2. However, for $D_{2}^{\prime }⩾0$, the nanosystem does not experience compensation temperatures, which is possible because the influence of $D_{1}^{\prime }=-1.9$ combined with $J_{1}=-1$ is significantly larger than the influences of $D_{2}^{\prime }⩾0$. This effect could be associated with or influenced by the nanoparticle size. On the other hand, since as the values of $D_{2}^{\prime }$ increase markedly, the same behavior is observed in $M_{T}$, we might suggest that this is independent of the positive increase in $D_{2}^{\prime }$.

**Figure 5:**
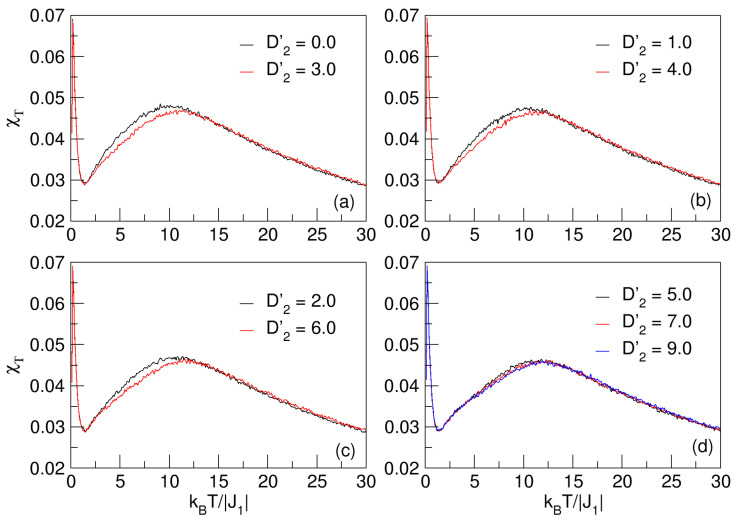
Magnetic susceptibility as a function of temperature for $0⩽D_{2}^{\prime }⩽9$ and $D_{1}^{\prime }=-1.9$. In (**a**–**d**), a slight shift of the flattened maximum of the curves toward the higher temperature region is observed. The maxima of these smooth $\chi_{T}$ curves represent a pseudo-transition or a pseudo-critical temperature.

Similarly for the $M_{T}$ curves in Figure 2, the effect of $D_{2}^{\prime }$ on the nanosystem also does not evidence critical temperatures at $M_{T}$; that is, pseudo-critical transitions are also present, a phenomenon that was extensively explained when the effects of $D_{1}^{\prime }$ were studied in Section 3.1. In Figure 5, the behavior of the magnetic fluctuations as a function of temperature in the curves of $\chi_{T}$ vs. *T* can be appreciated, where again there are smooth maximum peaks in the vicinity of the pseudo-critical temperatures, which increase with increasing $D_{2}^{\prime }$.

**Figure 6:**
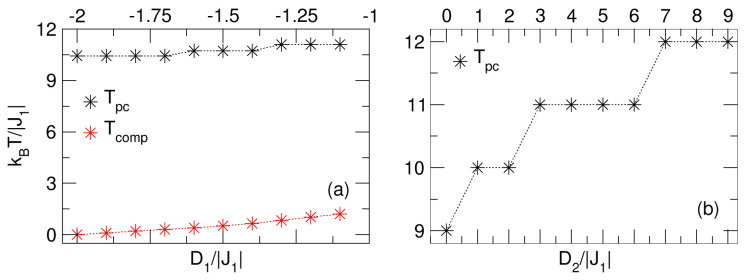
Summary of the behavior of the pseudo-critical ($T_{pc}$) and compensation ($T_{comp}$) temperatures: (**a**) due to the influence of the $D_{1}^{\prime }$ field with $D_{2}^{\prime }=1$, and (**b**) due to the effects of the $D_{2}^{\prime }$ anisotropy with $D_{1}^{\prime }=-1.9$. In both cases, $T_{pc}$ is increased by the action of the corresponding crystal field. The dotted lines are a guide for the eye.

A summary of the influences of $D_{1}^{\prime }$ and $D_{2}^{\prime }$ on the temperatures $T_{pc}$ and $T_{comp}$ are detailed in Figure 6a,b. It is observed that $T_{pc}$ and $T_{comp}$ experience a slight growth with the negative decrease in the $D_{1}^{\prime }$ crystal field (see Figure 6a). Meanwhile, $T_{pc}$ grows gradually with the positive increase in $D_{2}^{\prime }$, indicating a larger effect of the anisotropy of the Fe spin sublattice (Q-type), as shown in Figure 6b.

### 3.3. $J_{1}$–$D^{\prime }$–$h^{\prime }$ Model: Effects of $D^{\prime }$

**Figure 7:**
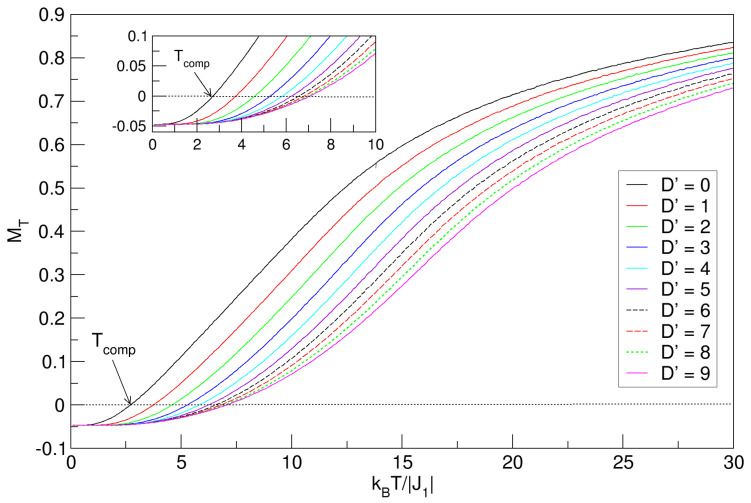
Effects of the $D^{\prime }>0$ average crystal field on the total magnetization of the quasi-spherical FeCo nanoparticle in the absence of external field $\left(h^{\prime }=0\right)$. In the interval $0⩽D^{\prime }⩽9$, $M_{T}$ exhibits compensation points ($T_{comp}$), which increase with $D^{\prime }$. The compensation phenomenon is enlarged in the inset above.

In this section, we include changes with respect to the previous model (Section 3.1 and Section 3.2). Consider now a $D^{\prime }$ average anisotropic field for the entire lattice, between $D_{1}^{\prime }$ and $D_{2}^{\prime }$, and consider the calculation of the physical variables $M_{T}$ and $\chi_{T}$ as a function of temperature, initially in the absence of a magnetic field ($h^{\prime }=0$), and then in the presence of a fixed external field with $h^{\prime }=-0.5$. The finite-temperature phase diagram of $M_{T}$, in the absence of external fields, is shown in Figure 7, for $0⩽D^{\prime }⩽9$. The $M_{T}$ curves present a constant value in the ground state magnetization ($k_{B}T/\left|J_{1}\right|=0$), for each value of the magnetocrystalline anisotropy, and their tendency is to increase with increasing temperature, until reaching an approximately constant saturation value for the higher temperature region. The constant value experienced by $M_{T}$ at T=0 could be explained by the fact that for positive anisotropies $D^{\prime }>0$, regardless of their value, the ground state configuration tends to stabilize, meaning all spins are aligned at a minimum magnetization and the system is oriented in the direction of easy magnetization.

The nanostructure undergoes compensation phenomena in the range $2.5⪅T_{comp}⪅7.5$, because the lattice spins tend to orient in the direction imposed by $D^{\prime }>0$, whereby the magnetization prefers to align itself along a specific axis of the crystal, usually the easy axis; meanwhile, the $J_{1}$ antiferromagnetic exchange, determining the general magnetic order in addition to the phase stability of the nanosphere, exerts internally a strong opposition, and many magnetic moments are reoriented to the point that at a specific value of the temperature, the sublattices are compensated—i.e., they have the same magnetization value with opposite signs. It has been proved in some cases of mixed-spin magnetic systems that first-neighbor exchange interactions and anisotropic couplings, represented by the parameters $J_{1}$ and $D^{\prime }$, are responsible for the origin of compensation points in these systems [38,39,40]. The trend of $T_{comp}$ indicates an increase with respect to the increase in the $D^{\prime }$ field. The enlarged inset shows the region where the compensation phenomenon occurs. Analogous to Figure 2 and Figure 4, where the influences of the $D_{1}^{\prime }$ and $D_{2}^{\prime }$ fields on the magnetization behavior of the FeCo nanocrystal are shown, in Figure 7, $M_{T}$ reflects quite similar curves for $D^{\prime }=D_{1}^{\prime }=D_{2}^{\prime }$, which increase with temperature; in the latter figure, the system also maintains its magnetic stability, with the difference that in Figure 7, they start at T=0 with the nonzero magnetization ($M_{T}\neq 0$)—that is, the $D^{\prime }$ average anisotropy rearranges the spins in the nanosystem at low temperatures. The critical points for the system to pass to the paramagnetic phase are also not visible in this case, which is explained in Section 3.1.

**Figure 8:**
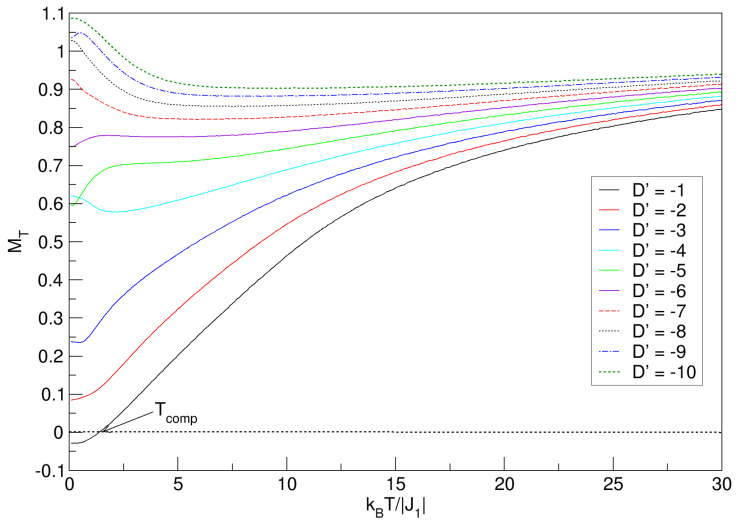
Effects of the $D^{\prime }<0$ average crystal field on the total magnetization of the quasi-spherical FeCo nanoparticle in the absence of external field ($h^{\prime }=0$). $M_{T}$ exhibits $T_{comp}$ only for $D^{\prime }=-1$. For large temperature values, each of the $M_{T}$ curves tends to a constant value.

An important aspect to consider to continue the thermomagnetic analysis of the FeCo nanoconfiguration is the effect of negative anisotropy ($D^{\prime }<0$) on the behavior of $M_{T}$. In Figure 8, by reversing the anisotropic direction, the imbalance in the sublattices can be noticed with the breaking of the compensation phenomenon; only one point prevails when $D^{\prime }=-1$ (the closest to $D^{\prime }=0$), with a lower value than all values of $T_{comp}$ for positive anisotropies. Interesting, too, is the diversity of ground-state values that arise when $D^{\prime }<0$. For $-6⩽D^{\prime }⩽-1$, curves increase with temperature, and in the range $-10⩽D^{\prime }⩽-7$, the magnetization decreases. In both ranges, it is observed that the curves tend to quasi-constant values for high temperatures. The consideration is that the type of behavior of the magnetization, in both $D^{\prime }$ ranges, is caused by the size of the nanosphere, which imposes confinement conditions, which undoubtedly affect the $J_{1}$ exchange energy; here, a non-uniform distribution of spins in the nanostructure can arise, generating a prevalence of anisotropic coupling over exchange. When the intensity of $J_{1}$ decreases, and spintronic (non-uniform) redistribution of domains in the nanomaterial appears, each time the anisotropy increases, many of these domains prefer to align along an axis that minimizes the total energy of the system. The superiority of the negative anisotropy intensity over the exchange energies makes the nanosphere have better magnetic stabilization; this is a property that can be appreciated for the highest values of $D^{\prime }$ in the interval $-10⩽D^{\prime }⩽-7$.

To complement the occurrence of the spectrum of $M_{T}$ values in the ground state, in addition to the phenomena already mentioned, negative anisotropies also generate other interesting physical situations in nanoscaled systems. Indeed, as in our study, when one has a ferrimagnetic exchange coupling ($J_{1}<0$) and $D^{\prime }$ is more negative ($-10⩽D^{\prime }⩽-7$), then the system will experience a strong preference for the spins to align in a specific direction, resulting in a higher magnetization in the ground state; thus, with increasing temperature, the thermal energy ($k_{B}T$) will reorient many random magnetic moments, reducing the magnetization. On the other hand, when $D^{\prime }$ is less negative, the preference for aligning the spins is weaker, resulting in a lower magnetization in the ground state. Interestingly, for the case of less negative anisotropies, the increase in temperature helps to increase $M_{T}$, since the thermal energy and thermomagnetic fluctuations cause some “local” energy barriers imposed by the $D^{\prime }$ field to be overcome, allowing more spins to align in the preferred direction and the nanostructure to become magnetized. All of the above leads to the conclusion that $D^{\prime }$ anisotropy effects tend to dominate especially at low temperatures.

**Figure 9:**
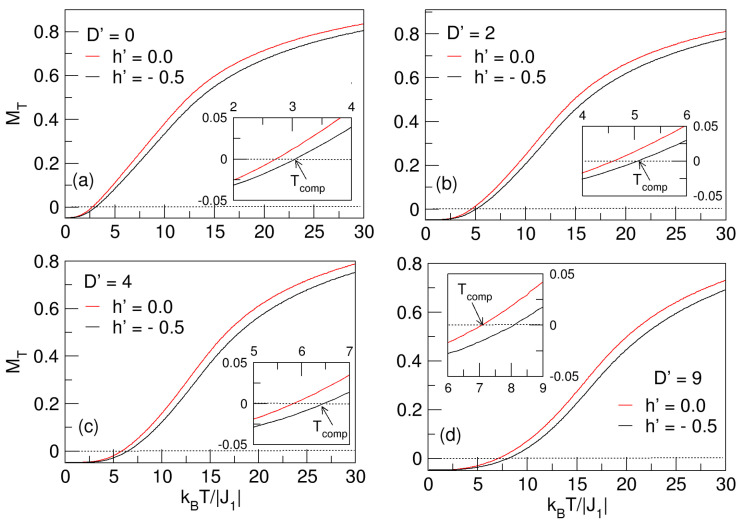
Comparison of the total magnetization behavior of the quasi-spherical FeCo nanosystem in the absence ($h^{\prime }=0$) and presence ($h^{\prime }=-0.5$) of an external magnetic field. The effect of the $D^{\prime }$ anisotropic field is noted at the compensation points ($T_{comp}$) highlighted in the inner insets of the figure. In (**a**), $T_{comp}$ exists even in the absence of $D^{\prime }$. In (**a**–**d**), the proportional increase in $T_{comp}$ with respect to $D^{\prime }$ is noticeable.

As interesting as the previous analyses is the study of the influence of an external magnetic field on the direction of the $J_{1}$ exchange interaction. Considering the same model $J_{1}$–$D^{\prime }$–$h^{\prime }$, let us subject the nanosphere to a $h^{\prime }=-0.5$ fixed field with $0⩽D^{\prime }⩽9$. In Figure 9a–d, we note the effect of $h^{\prime }$ on the nanosystem. The performed comparison of the behavior of $M_{T}$ for $h^{\prime }=0,-0.5$ is carried out, noting in the inner boxes a broadening of the compensation points. In this case, $T_{comp}$ is not destroyed because $J_{1}$ and $h^{\prime }$ keep the spins in a given antiferromagnetic direction; that is, the external field reinforces the existing of antiferromagnetism in the lattice by next-neighbor exchange effects. The $D^{\prime }>0$ magnetocrystalline anisotropy tries to counteract the exchange effect; competition does not unbalance the sublattices, but moves the compensation temperature toward slightly larger values, as noticed in the insets of Figure 9a–d. Due to the effect of $D^{\prime }>0$ many magnetic moments, especially from the spin states of ±2 values in Fe and ±3/2 in Co, the system tries to find an easy magnetization axis in the lattice and causes the shift of the compensation points. It is worth noting that another factor contributing to the shift of $T_{comp}$ is that $h^{\prime }$ slightly modifies the magnetization of the *A* and *B* sublattices, such that $M_{A}$ in the *S* spin sublattice decreases subtly, and $M_{B}$ in the *Q* spin sublattice increases. This implies that the external field is oriented parallel to the axes of easy magnetization.

**Figure 10:**
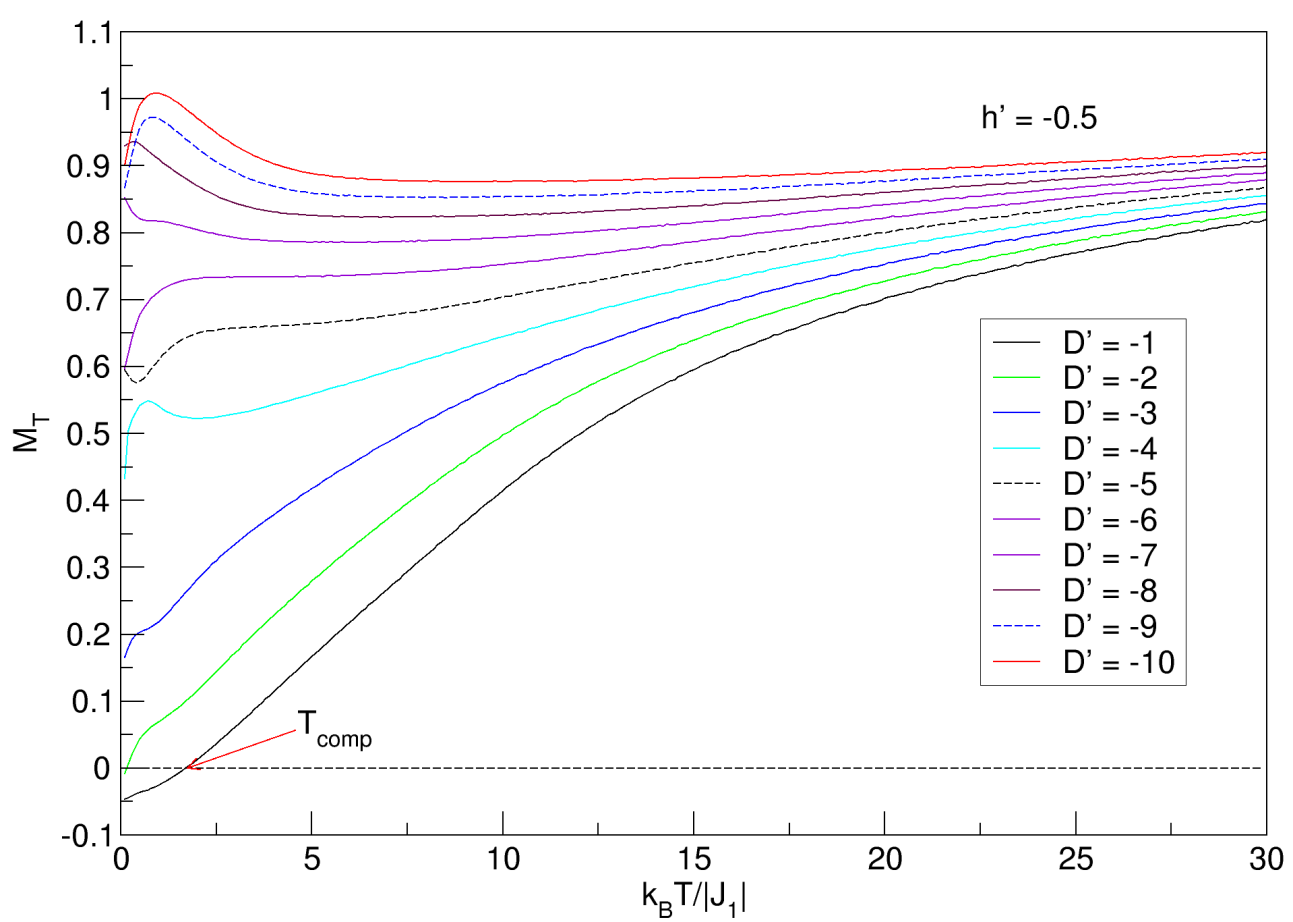
Effects of the $D^{\prime }$ average crystal field on the total magnetization of the quasi-spherical FeCo nanoparticle in the presence of an external field ($h^{\prime }=-0.5$) in the range $-10⩽D^{\prime }⩽-1$. $M_{T}$ exhibits $T_{comp}$ only for $D^{\prime }=-1$. For large temperature values, each of the $M_{T}$ curves tends to a quasi-constant value.

The effects of negative anisotropies and the external field $h^{\prime }=-0.5$ are seen in Figure 10. As in Figure 8, for $h^{\prime }=0$, the system experiences a variety of values in the ground state, but with smaller values of $M_{T}$. This reduction is due to the fact that for more negative anisotropies ($-10⩽D^{\prime }⩽-7$), the external field is perpendicular to the spin orientation in the interval $-10⩽D^{\prime }⩽-7$, so that a preference arises towards the weaker alignment, manifesting in this case an increase in magnetization with increasing temperature. For large values of $k_{B}T/\left|J_{1}\right|$, all curves tend to a quasi-constant value. It should be noted that the qualitative behavior of the $M_{T}$ curves with $h^{\prime }=-0.5$ in Figure 10 is similar to that shown in Figure 8 with $h^{\prime }=0$. A physical explanation of the behavior of magnetization as a function of temperature has been given in Section 3.1 and Section 3.2.

**Figure 11:**
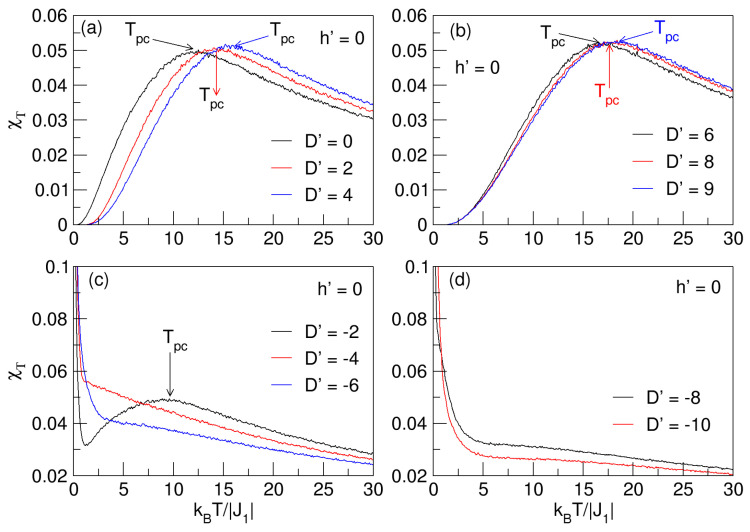
Total magnetic susceptibility as a function of temperature for $D^{\prime }⪌0$ in the absence of an external magnetic field ($h^{\prime }=0$). In (**a**,**b**) for $D^{\prime }⩾0$, the pseudo-critical temperatures ($T_{pc}$), represented in the “flattened” maxima of the curves, increase with increasing $D^{\prime }$. In (**c**,**d**), for $D^{\prime }<0$, only $T_{pc}$ is seen for $D^{\prime }=-2$.

**Figure 12:**
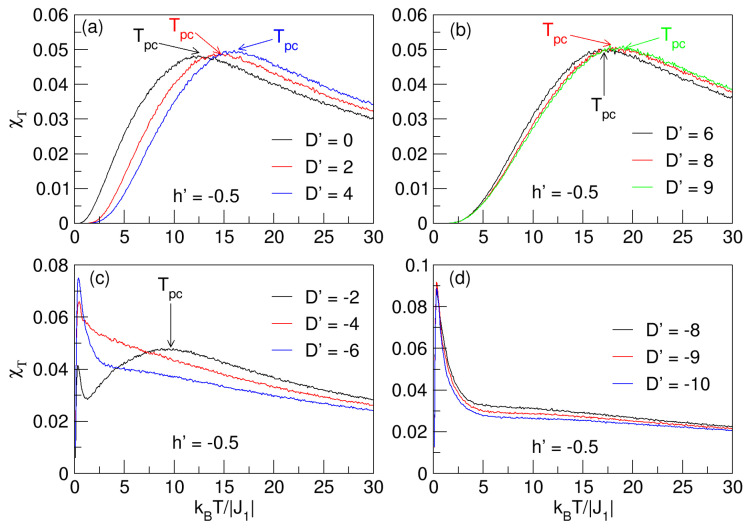
Total magnetic susceptibility as a function of temperature for $D^{\prime }⪌0$ in the presence of an external magnetic field ($h^{\prime }=-0.5$). In (**a**,**b**) for $D^{\prime }⩾0$, the pseudo-critical temperatures ($T_{pc}$), represented in the “flattened” maxima of the curves, increase with increasing $D^{\prime }$. In (**c**,**d**), for $D^{\prime }<0$, only $T_{pc}$ is seen for $D^{\prime }=-2$, and at low temperatures, there are non-critical peaks resulting from the thermal rearrangement of the spins in the nanoparticle.

For the analysis of the magnetic fluctuations and the approximate location of the continuous phase transitions of the nanosphere, i.e., the $T_{pc}$ pseudo-critical temperatures, considering the behavior of the total magnetic susceptibility $\chi_{T}$ in the absence and presence of a magnetic field ($h^{\prime }=0,-0.5$) and in the range $-10⩽D^{\prime }⩽9$, the analysis information is displayed in Figure 11 and Figure 12.

The behaviors of the $\chi_{T}$ curves in Figure 11a,b display flattening at their maxima, as do the susceptibility curves in Figure 3 of Section 3.1; therefore, the transition to the paramagnetic phase cannot be accurately determined when it comes to pseudo-critical temperatures ($T_{pc}$), as explained in Section 3.1. As $D^{\prime }$ anisotropy increases, $T_{pc}$ also increases. A sign of the increase in the pseudo-critical temperature is reflected in a higher magnetic stability of the system, which is present when the anisotropy increases. As for Figure 11c,d for negative anisotropies, there are a “smooth” maxima for a value of $D^{\prime }$ close to zero ($D^{\prime }=-2$), but as the anisotropy increases to larger negative values ($D^{\prime }⟶-10$), the magnetic fluctuations increase, and $\chi_{T}$ becomes much smaller.

**Figure 13:**
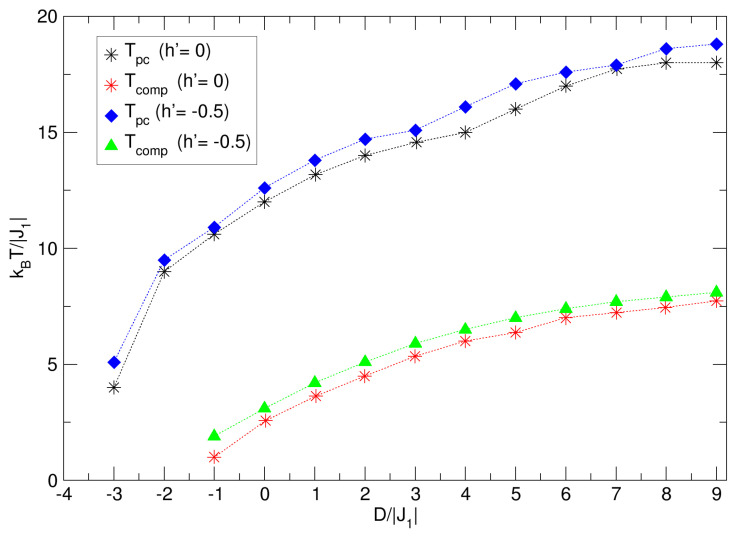
Summary of the behavior of the pseudo-critical ($T_{pc}$) and compensation ($T_{comp}$) temperatures, in the absence and presence of an external magnetic field, due to the influence of the $D^{\prime }$ field. In both cases, $h^{\prime }=0$ and h´=−0.5, it is noted that $T_{pc}$ and $T_{comp}$ are proportional to $D^{\prime }$. In addition, the presence of the magnetic field causes $T_{pc}$ and $T_{comp}$ to increase slightly.

The analysis of the effect of a fixed negative field ($h^{\prime }=-0.5$) is detailed in Figure 12. For both cases, $D^{\prime }⩾0$ (Figure 11a,b and Figure 12a,b) and $D^{\prime }<0$ (Figure 11c,d and Figure 12c,d), the behavior is analogous to the case in Figure 11; that is, $T_{pc}$ grows with increasing $D^{\prime }⩾0$ (see Figure 12a,b) and decreases when $D^{\prime }<0$ (see Figure 12c,d). The difference lies in the fact that the presence of $h^{\prime }$ on the nanosphere causes the $T_{pc}$ to increase slightly with respect to the $h^{\prime }=0$ case. A summary analysis of the behavior of the temperatures $T_{pc}$ and $T_{comp}$, deduced from Figure 7, Figure 8, Figure 9, Figure 10, Figure 11 and Figure 12, is detailed in Figure 13 for $h^{\prime }=0,-0.5$, corroborating what has already been explained.

### 3.4. Hysteresis Behavior at Different Temperatures

Magnetic nanoparticles present several characteristics that generate phenomena not often present in massive materials; because of this particularity in their physical behavior, it is important to analyze them under certain magnetic interactions, which bring out the phenomenological richness that nanostructured materials possess. In the following, we will analyze the hysteretic behavior of the nanosphere for different temperatures, when it is influenced by the $D_{1}^{\prime }$ and $D_{2}^{\prime }$ anisotropic fields of the sublattices that compose it, and by the $D^{\prime }$ average field thereof.

#### 3.4.1. Influences of Anisotropy: $D^{\prime }$

In this section, we will analyze what happens when we equalize the anisotropies, estimating that $D_{1}^{\prime }=D_{2}^{\prime }=D^{\prime }$ represents the average crystal field of the whole nanoscaled system. We will study the influences of the $D^{\prime }$ total anisotropy on the nanoparticle for positive and negative values.

**Figure 14:**
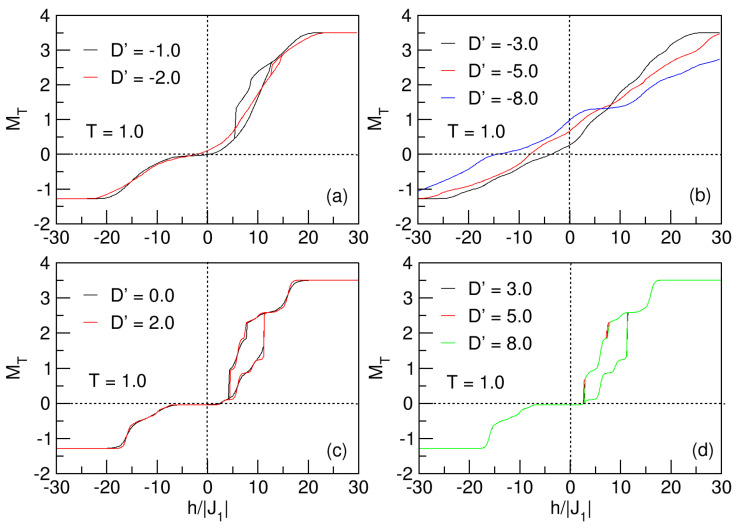
Hysteretic behavior of the quasi-spherical FeCo nanoparticle for $D^{\prime }⪋0$ and T=1. In (**a**), the nanosystem experiences the exchange bias phenomenon, for $D^{\prime }=-1,-2$. In (**b**), the negative increment of $D^{\prime }$ transfers the nanoparticle to the superparamagnetic phase. For $D^{\prime }⩾0$ in (**c**,**d**), the system undergoes exchange bias, with loops of approximately constant areas.

Initially, we study how $D^{\prime }$, in the ranges $-8⩽D^{\prime }⩽-1$ and $0⩽D^{\prime }⩽8$ for T=1, influences the hysteresis behavior of the FeCo nano-ferrimagnet for $J_{1}=-1.0$. In Figure 14a,c,d, the nanosystem exhibits exchange bias, a phenomenon generally caused by an interfacial exchange between two couplings, one ferromagnetic and the other antiferromagnetic, such that for $T_{c}<<T_{N}$, a kind of unidirectional exchange anisotropy ($D_{exc}$) arises in the nanosystem, which acts as another field, and when it is in the same direction as the $h^{\prime }$ applied field, it shifts the hysteresis loop. For magnetic nanoconfigurations, the effects due to exchange anisotropy in their magnetization are reflected by horizontal or vertical displacements of the hysteresis loops, which are asymmetric with respect to the $h^{\prime }=0$ axis, or else a widening of the loop occurs [41]. In the hysteretic processes of nanostructured magnetic systems, as the external magnetic field increases, the magnetic domains of the material are reoriented in the direction of the field, so that their magnetization increases progressively, gradually breaking the walls of these domains until saturation is reached (formation of a monodomain). However, often, in addition to the external field, additional interactions can be considered in the Hamiltonian that defines the nanostructure, which implies many times the appearance of interesting physical phenomena reflected in the $M_{T}$ vs. $h^{\prime }$ curves. For example, the influence of these intrinsic couplings of the nanoconfiguration could cause sudden jumps or drops that destroy the apparent continuity or smoothness of the curves. These abrupt jumps are due to the displacement of the domain walls by the effects of the internal parameters of the nanoparticle; it is possible that for some values, this wall breaking is not continuous, but increases or decreases the intensity of the magnetization of the system.

The research concerns of this paper limit us to taking into consideration exchange, anisotropic, and external field couplings. For negative anisotropies ($D^{\prime }<0$), the magnetization ($M_{T}$) of the nanosystem tends to be oriented towards the direction of the hard axis; consequently, the negative intensity of $D^{\prime }$ increases, and it is more difficult to magnetize—i.e., to reach saturation ($M_{T}=M_{s}$), larger external magnetic fields are required. This situation results in a slower displacement of the domain walls and smoother magnetization curves (no abrupt jumps). On the contrary, when $D^{\prime }>0$, the tendency of the magnetization in the nanoparticle is oriented towards the easier axis, so that for large positive anisotropies, it is easier to magnetize the material, accelerating the formation of the monodomain when $M_{T}=M_{s}$, due to faster displacements of the walls. The above leads to the appearance of jumps in the magnetization curves as a consequence of the gradual decrease in these domains. This phenomenology is explained below, through the hysteresis curves of the nanoparticle for T=1 in the Figure 14.

Figure 14a shows that in the range of $-30⩽h^{\prime }⩽-20$, the nanoparticle exhibits saturation, since the external field is not yet able to disrupt the lattice to change the magnetization of the nanosystem. Meanwhile, for $-20⩽h^{\prime }⩽0$, the external field reorients the spins of the magnetic domains, then the magnetization cancels out for $h^{\prime }=0$ in the case of the $D^{\prime }=-1$ curve and exhibits a very small coercive field and remanence (different from zero) for the $D^{\prime }=-2$ case. By positively increasing the external field, $0<h^{\prime }⩽20$, the magnetization of the system increases smoothly until saturation is reached for $h^{\prime }⩾20$ fields. Upon demagnetizing the system, it can be observed that for small anisotropies ($D^{\prime }=-1$), the system exhibits irreversibility, as manifested by the hysteresis loop in the first quadrant. This is a result of anisotropy, interactions between the ferromagnetic and antiferromagnetic sublattices, and surface effects. This phenomenon is known as exchange bias. In Figure 14b, what was previously explained about the influence of $D^{\prime }<0$ in the magnetization of the material can be seen; the figure highlights how the coercive field and the remanence increase as the magnitude of the anisotropy increases, making it more difficult to magnetize the nanoparticle to saturation. On the other hand, the effect of $D^{\prime }>0$ in Figure 14c,d biases the orientation of the nanoparticle magnetization toward an easy axis, whereby the nanosystem exhibits discontinuities (sudden jumps) due to abrupt displacements of the domain walls, as noticed for $h^{\prime }≃4.2$ and $h^{\prime }≃11.2$ in Figure 14c, as well as for $h^{\prime }≃2.6$ and $h^{\prime }≃11.2$ in Figure 14d; this situation gives rise to the presence of exchange bias in the nanoparticle.

**Figure 15:**
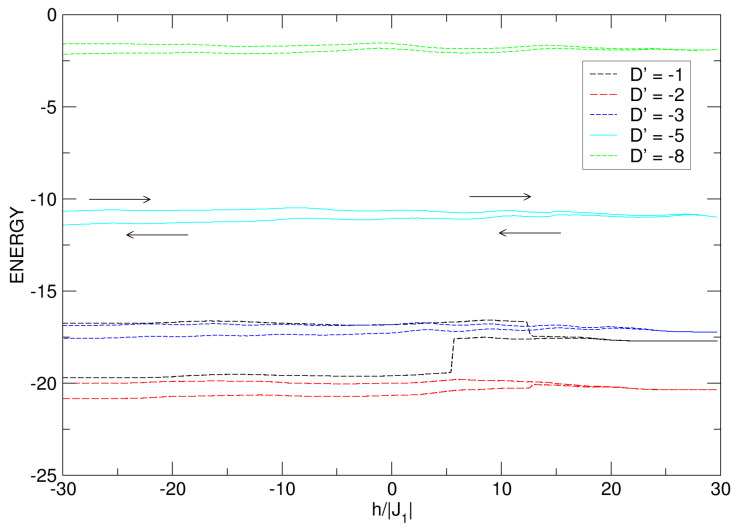
Internal energy of the quasi-spherical FeCo nanoparticle under the effects of the $D^{\prime }<0$ anisotropic field. The negative increase in $D^{\prime }$ leads to a gradual decrease in the energy. The arrows indicate the energetic process, which starts at the top of each curve in the range $-30⩽h^{\prime }⩽30$.

As is known, anisotropy introduces a directional preference for spin orientation, creating an energy barrier that the spins must overcome to change direction; as this increases, for $D^{\prime }>0$, the energy of the system increases, while for $D^{\prime }<0$, the energy of the system decreases. In Figure 14b, for cases with no hysteresis loops ($D^{\prime }=-3,-5,-8$), it is as if there is a threshold value of $D^{\prime }<0$ ($D^{\prime }=-3$) above which the loops are destroyed and possible superparamagnetic states are experienced in the nanoparticle. Thus, as the anisotropy increases negatively from this threshold value ($D^{\prime }\to -8$), the absolute value of the energy decreases, along with ΔE, as seen in the curves of Figure 15. It is relevant to emphasize that the hysteretic behavior of a magnetic system is associated with changes in its internal energy during a complete cycle of magnetization and demagnetization. For a better understanding of this behavior, the energy curves presented for the nanoconfiguration for the same values of the anisotropy are shown in Figure 14.

For negative crystal fields, for $D^{\prime }<0$, in Figure 15, a noticeable change in energy can be observed: comparing the curves for $D^{\prime }=-1$ and $D^{\prime }=-2$, a larger change is noticed when $D^{\prime }=-1$ (interval −19.7≈E≈−16.7). The energy curve for $D^{\prime }=-1$ and $h^{\prime }=-30$ starts with a quasi-constant value from E=−16.7 until experiencing an abrupt drop at approximately $h^{\prime }≃12.5$, and then ends after a half cycle when $h^{\prime }=30$. Once the external field is reversed, the nanosphere presents another sudden drop for $h^{\prime }≃5.5$, and continues its irreversible process with a quasi-constant value of E≃−19.7, until completing the cycle when $h^{\prime }=-30$. This indicates that the difference between the energy values when the nanosystem makes the complete hysteretic cycle is ΔE≃−16.7−(−19.7)=3. In an analogous process for $D^{\prime }=-2$, it is noted that the increase in anisotropy generates a smaller energy difference (ΔE≃−20−(−20.8)=0.8) by developing the whole cycle in the range $-30⩽h^{\prime }⩽30$. All of the above is in agreement with the hysteresis curves in Figure 14a, where a smaller area is noted for the loop associated with $D^{\prime }=-2$. The intensity of these values of $D^{\prime }$ is so small that the system is able to overcome the anisotropic barrier to become magnetized on the hard axis and form a single loop for this value of the anisotropic field, but with different areas.

**Figure 16:**
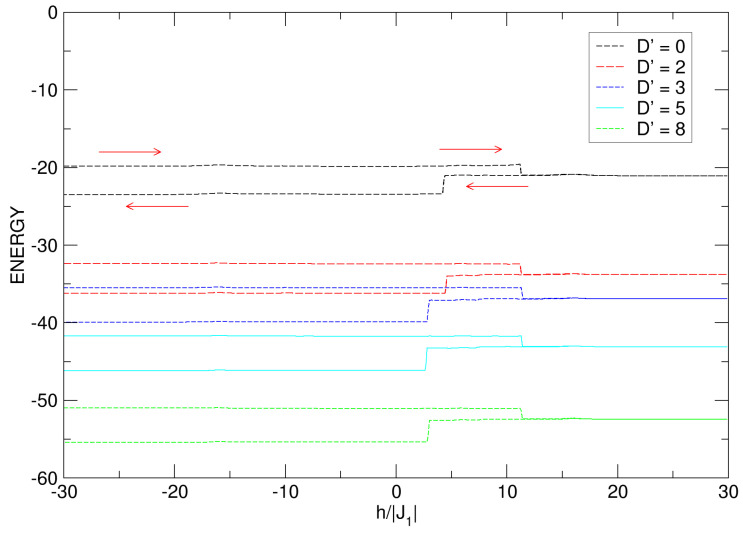
Internal energy of the quasi-spherical FeCo nanoparticle under the effects of the $D^{\prime }⩾0$ anisotropic field. The positive increase in $D^{\prime }$ leads to a gradual increase in the energy. The arrows indicate the energetic process, which starts at the top of each curve in the range $-30⩽h^{\prime }⩽30$.

For $D^{\prime }⩾0$, in Figure 16, as expected and according to the results observed in the curves of $M_{T}$ vs. $h^{\prime }$, there is variation in the energy with changes approximately equal (ΔE) for all cases; in addition, sudden jumps in the irreversible process of the energy cycle are noticed, for the same values of $h^{\prime }$ where such jumps are presented for the magnetization (see Figure 14c,d). It should be noted that as $D^{\prime }$ increases, the absolute value of the energy increases.

#### 3.4.2. Influences of Anisotropy: $D_{1}^{\prime }$

As interesting as the effects of an average crystal field $D^{\prime }$ for the nanosystem at hand turns out to be the influence of each of the single-ion anisotropies of the sublattices composing the nano-ferrimagnet. We begin in this section, Section 3.4.2, an analysis of the behavior of the magnetization when the effect of the anisotropy of the sublattice of Co atoms, $D_{1}^{\prime }$, with spins S=±3/2,±1/2 is taken into account.

**Figure 17:**
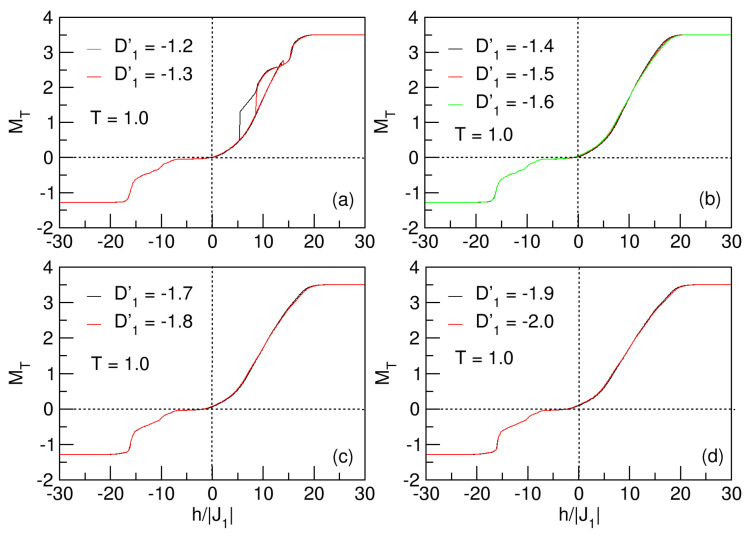
Hysteretic behavior of the quasi-spherical FeCo nanoparticle, for $D_{1}^{\prime }<0$ and T=1. In (**a**), the nanosystem experiences the exchange bias phenomenon, for $D^{\prime }=-1.2,-1.3$. There is a threshold value of $D_{1}^{\prime }$ in the range $-1.4<D_{1}^{\prime }<-1.3$, at which the nanoparticle enters the superparamagnetic phase, as shown in (**b**–**d**).

In Figure 17a, the nanosystem exhibits an asymmetric loop to the right of the $h^{\prime }=0$ axis for each of the values of $D_{1}^{\prime }$, which decreases with increasing |$D_{1}^{\prime }$|.

As in Figure 14, when considering the $D^{\prime }=D_{1}^{\prime }=D_{2}^{\prime }$ average anisotropy, in Figure 17, there is also a threshold value, $D_{1}^{\prime }=-1.4$, at which the hysteresis loops are destroyed, and the system enters the superparamagnetic phase. In this case, this value is smaller because there is competition with the anisotropy $D_{2}^{\prime }=1$. Similarly, a qualitatively analogous behavior to that analyzed for $D^{\prime }<0$ is observable.

There are some factors that lead the system to the exchange bias phenomenon, which we consider below. Initially, the $D_{2}^{\prime }>0$ crystal field tries to order the *Q*-type spin lattice (*B* sublattice with Fe atoms) by orienting its magnetization towards an easy axis. This apparent order of the *B* sublattice is counteracted by the action of the $J_{1}$ antiferromagnetic exchange to the first neighbors, and the orientation of the magnetization of the *A* sublattice spins (Co atoms) towards a difficult axis, a product of the $D_{1}^{\prime }<0$ anisotropic field. This anisotropy exchange competition between the parameters of the Hamiltonian reduces the hysteresis loops, and at low temperatures, the system is superparamagnetic, as shown for $D_{1}^{\prime }<-1.3$ values, as expected for this type of nanoparticle. For the range $D_{1}^{\prime }<-1.3$, the thermomagnetic evolution of the nanostructure is independent of the anisotropic effect, since superparamagnetism is still constantly present.

#### 3.4.3. Influences of Anisotropy: $D_{2}^{\prime }$

**Figure 18:**
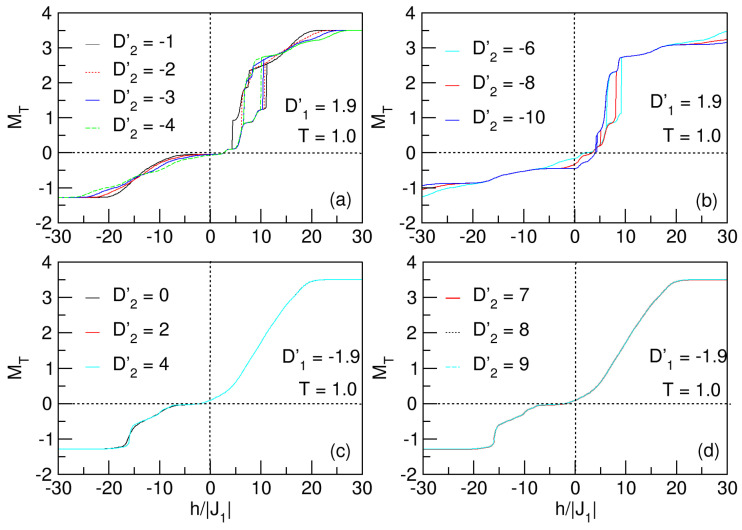
Hysteretic behavior of the quasi-spherical FeCo nanoparticle, for $D_{2}^{\prime }⪋0$ and T=1. In (**a**,**b**), for $D_{1}^{\prime }=1.9$, the nanosystem experiences the exchange bias phenomenon in the ranges $-4⩽D_{2}^{\prime }⩽-1$ and $-8⩽D_{2}^{\prime }⩽-6$, respectively. The area of the loops is ostensibly reduced with the negative increment of $D_{2}^{\prime }$, until passing to the paramagnetic phase when $D_{2}^{\prime }=-10$. For $D_{2}^{\prime }⩾0$ and $D_{1}^{\prime }=-1.9$. In (**c**,**d**), the system undergoes the superparamagnetic phenomenon.

The influence of the $D_{2}^{\prime }$ crystal field on the hysteretic behavior of the nanosystem is shown in Figure 18a,b. Analogous to the previously studied cases on the effect of $D_{1}^{\prime }$ and $D^{\prime }$ on the magnetization behavior, the influence of $D_{2}^{\prime }$ on the hysteresis of the system also reflects the exchange bias phenomenon, with a decrease in the loop area when the crystal field grows negatively.

Similarly, a threshold value of $D_{2}^{\prime }=-10$ (much larger than the previous ones), at which the system transitions to the superparamagnetic phase, is also seen. Figure 18a,b also show that there is a prevalence of $D_{1}^{\prime }$ anisotropy to keep the lattice more ordered. This reflects the need for a larger crystal field, oriented on the hard axis ($D_{2}^{\prime }=-10$), to disorder the lattice, and make the transition to the superparamegnetic phase. The dominance of the crystal field associated with the sublattice of Co atoms ($D_{1}^{\prime }<0$) on the nanosystem can be seen in the same way in the curves of Figure 18c, since although the anisotropy $D_{2}^{\prime }>0$ tends to orient the spins towards the easy axis with values from zero to four, it is not enough to overcome the energy barrier caused by the anisotropy $D_{1}^{\prime }$, since superparamagnetism is observed in all cases.

## 4. Discussion

The most significant thing to highlight in this research is how, through computational simulations, the qualitative behavior of various nanostructured physical systems can be obtained, which is reflected in the different phenomena they experience. These simulations help to support and complement several experimental investigations, allowing for predicting the behavior of systems under different conditions. Likewise, they support the search for and design of new materials with specific properties before attempting to synthesize them. In our case, this paper deals with a quasi-spherical nanoparticle in the presence of exchange, an external field, and, above all, anisotropic interactions. Among the phenomena found due to the effects of these interactions on the nanoparticle, it is pertinent highlight pseudo-critical and compensation temperatures, as well as hysteresis loops and superparamagnetism at low temperatures.

Comparing the results of this paper with previous work on nanostructured systems, Ahsan and Singh [42] in their work on Atomistic simulation study of FeCo alloy nanoparticles reported critical temperatures for different concentrations of the alloy; in contrast to their research, we only found pseudo-critical temperatures, which do not clearly reflect the ferri-paramagnetic phase transition. Another important aspect to highlight is the finding of compensation and exchange bias phenomena in this paper, which were not reported in the [42]. On the other hand, Kumari et al. [43] experimentally investigated magnetocrystalline anisotropy and exchange bias phenomena through the interface effects of nanocrystalline FeCo; they demonstrated, as achieved in this research, that the behavior of magnetization as a function of temperature does not show critical temperatures—i.e., the qualitative behavior of the magnetization curves in [43] (see Figure 5a of [43]) is analogous to that reported in our work in Figure 2, Figure 4, and Figure 9. Another experimental demonstration that corroborates our findings on how $M_{T}$ increases with thermal energy is established in work on the exchange bias effect in FeCo nanoparticles reported by Potpattanapol et al., who fabricated three ${\mathrm{Fe}}_{1-x}{\mathrm{Co}}_{x}$ nanoparticles using the hydrothermal method, where XRD studies determined that the structures of the first two nanoparticles were body-centered cubic (BCC) [44]. Potpattanapol et al. measured their magnetic properties through FC and ZFC magnetization curves (see Figure 3a,b of [44]) in order to study the exchange bias effects that cause a horizontal shift of the hysteresis loops, also reflected in this research. It is worth noting that as in this paper, in [44], the researchers posit that the differences between the FC and ZFC curves of the three FeCo nanoparticles are caused by the energy barriers resulting from the magnetic anisotropies in the three samples.

It should be noted that, in the present work, we used different values of internal couplings, such as the crystal fields $D_{1}^{\prime }$, $D_{2}^{\prime }$, and $D^{\prime }$, which allowed us to demonstrate their influence on the aforementioned phenomena. For example, the effect of $D_{1}^{\prime }$ generated pseudo-critical temperatures and compensation points in the system, while for the application of $D_{2}^{\prime }$, we only found $T_{pc}$. By averaging the anisotropies ($D_{1}^{\prime }=D_{2}^{\prime }=D^{\prime }$), the system experienced $T_{comp}$ and $T_{pc}$. Similarly, this study also permitted us to analyze the hysteretic behavior of the nanoparticle for various values of $D_{1}^{\prime }$, $D_{2}^{\prime }$, and $D^{\prime }$ in the easy and hard directions of the magnetization of the nanosystem. Our findings highlight the importance of computational simulations in the investigation of the thermomagnetic behavior of these nanostructures, as a complement to experimental investigations.

This discussion could be extended by complementing this work with other research in which new physical factors are involved. For instance, the influence of intrinsic parameters such as surface anisotropy might be studied, and future studies may consider these interactions under a different Hamiltonian, as in the case of the Heisenberg one that defines the nanosystem.

## Data Availability

The original contributions presented in this study are included in the article. Further inquiries can be directed to the corresponding author.
